# Editorial: National health services: Efficiency, welfare and economy

**DOI:** 10.3389/fpubh.2022.1095825

**Published:** 2022-12-02

**Authors:** María del Carmen Valls Martínez, José-María Montero, Annibale Biggeri

**Affiliations:** ^1^Mediterranean Research Center on Economics and Sustainable Development, Economics and Business Department, University of Almería, Almería, Spain; ^2^Department of Political Economy and Public Finance, Economic and Business Statistics, and Economic Policy, University of Castilla-La Mancha, Toledo, Spain; ^3^Department of Cardio-Thoracic-Vascular Science and Public Health, University of Padua, Padua, Italy

**Keywords:** national health services, efficiency, welfare, economy, health economics, health policy, life expectancy, mortality

The national health service (NHS) forms a crucial part of a country for improving the health and lives of its citizens (1, 2). It contributes toward increasing life expectancy and improving the quality of life of the population and allows for better control of mortality rates resulting from various diseases. In addition, it is a key element in promoting social equity and improving the efficiency of a country's economy. Health managers face significant challenges, such as the growing demand for higher quality service, the increase in life expectancy leading to population aging, rising costs due to advances in medical technology, and limited and scarce resources, especially as a result of the economic crisis (3–5). Nowadays, it is also necessary to consider the impact of the COVID-19 pandemic, which further complicates the context (6).

The quality of an NHS differs according to the country and geographical area in question (7, 8). It is essential to display the NHS's strengths, identify its weaknesses, and provide helpful information for practitioners, administrators, politicians, and citizens. The control of medical procedures, action protocols, cost management, and resource allocation must be analyzed from a purely medical and managerial perspective. The COVID-19 pandemic has highlighted how differences in management lead to very different health outcomes (9, 10). While some health systems have been able to manage the situation effectively, others have experienced major collapses at the most difficult times, resulting in higher mortality, more negative consequences for survivors, and a deepening economic crisis. The wellbeing and health of its population should be the primary objective of a country. Moreover, health resources management is essential for reducing inequalities in the population's health status and providing a stable and robust economy.

This Research Topic has contributed to providing empirical evidence in the field of health management as well as knowledge for enhancing both the efficiency of an NHS and the welfare of a country's population through research conducted worldwide, specifically in China, Japan, the United States, and Europe.

Shi et al. have revealed spatial and temporal differences in health expenditure efficiency in China based on the background of the COVID-19 pandemic. They used a meta-frontier data envelopment analysis (DEA) based on the number of health workers and beds in medical institutions as well as per capita fiscal health expenditure as the input variables, and GDP and life expectancy as the output variables. The results show a large difference between the eastern and western regions of China, with the former having the most efficient technical level of efficiency.

Guo et al. evaluated unified healthcare efficiency in China using a meta-frontier non-radial directional distance function analysis. The number of health technicians and beds were used as inputs, while the number of outpatient and inpatient visits, as well as the maternal, perinatal, and contagious mortality rates were used as outputs. These authors determined how efficiency is linked to technological progress, such that the more technologically developed regions have a more evolved healthcare system. In addition, the less developed areas follow the path of the more developed regions to reduce the efficiency gap.

Based on a non-parametric additive model, Wang Y. et al. established that the amount of public health spending is crucial not only for the survival and quality of life of a population but also for economic development. Therefore, especially during periods of economic crisis, governments should increase the percentage of GDP allocated to health resources to improve the health of human capital and, consequently, the performance of the economy.

Yang et al., using the two-stage network DEA model, showed how the efficiency of the Chinese healthcare system has increased, but also that there are notable differences between different regions. The factors that most affect efficiency are economic development, fiscal decentralization, and the old-age dependency ratio. Efficiency can be improved by establishing mechanisms for sharing resources and forming medical alliances between the most and least efficient areas. Governments should encourage technological innovation in the medical field and also increase technical medical research. It is also important to implement training programs that improve the skills of health technicians, further the education of resident physicians, and increase the population's health literacy.

Yin et al. analyzed efficiency in Chinese county hospitals based on DEA and a fuzzy-set qualitative comparative analysis. A combination of many factors determines efficiency. In this sense, achieving efficiency requires structural optimization, capacity enhancement, and government support. In contrast, inefficiency is determined by insufficient capacity, aggressive expansion, and poor decision-making.

Chen et al. studied the case of China and highlighted the difficult work performed by village doctors in developing countries, which often causes job burnout and turnover intention. Governments should develop policies to improve the situation of these doctors, such as reducing their workload by employing more doctors, training students for this particular position, and improving the harsh working conditions.

Life expectancy can be considered to be the fundamental determinant of wellbeing. Therefore, it is a critical element of government policies for achieving the United Nations Sustainable Development Goals. However, life expectancy differs greatly between countries, as well as between regions within the same country. In this vein, Valls Martínez et al. have revealed the inequalities in regional wellbeing arising from public healthcare expenditure policies in Spain. First, using a PLS-SEM model, the authors showed how public health spending has a decisive influence on the population's wellbeing as measured by life expectancy. Second, based on a hierarchical cluster analysis and a principal component analysis, they established a clear division of the country into areas according to the overall health of their citizens as well as basic spending. Therefore, public health spending policies make a difference even within the same country and are decisive for the wellbeing of citizens.

Moga Rogoz et al. have shown the positive effects of economic freedom and education on life expectancy in the new EU member states. Economic freedom leads to adequate legal structures, sound monetary and fiscal policies, financial development, trade liberalization, innovation, and competitiveness, which implies greater wellbeing. Moreover, better-educated individuals are more likely to adopt healthier lifestyles and have greater access to higher-paying jobs, allowing them to use improved health services. Analyzing the case of China, Wang S. et al. established that fiscal policy is a fundamental macroeconomic tool for boosting economic development, especially in times of crisis, such as that resulting from the COVID-19 pandemic. The expansionary fiscal spending policy failed in the economic control of the crisis, with the effectiveness of the revenue-based fiscal policy being much faster.

Carrasco-Aguilar et al. analyzed the literature on the Affordable Care Act in the United States, colloquially known as Obamacare. This law was intended to allow all U.S. citizens access to healthcare services at a time when most were provided by the private sector, representing a change in the country's mentality toward free and universal healthcare. This was a significant reform, with legal, political, economic, administrative, and health repercussions, and has brought about significant progress in preventing and treating illnesses. However, Yuda, in a study of Japan, demonstrated that free health care entails an excessive use of public health services, which can be moderated by establishing a co-payment system.

On the other hand, Hao et al., through an evaluation of a program for controlling hypertension in China, showed how public programs for the prevention, control, and treatment of certain illnesses have proven to be efficient. In this vein, Bosch-Frigola et al. (a), in a study on diabetes mellitus in the different European healthcare systems, showed how an increased awareness among the population of this disease, as well as improvements in national health plans, can improve the quality of life of patients, influencing the management of the disease and healthcare expenditure. The multiplier effect of specific prevention campaigns must also be considered. For example, considering the direct relationship between certain psychopathologies and increased rates of smoking, Nieto-González et al., showed that campaigns aimed at the prevention of mental illness may reduce tobacco consumption among patients. Finally, in a study conducted by Bosch-Frigola et al. (b) on diabetes mellitus in Spain, it has become clear how access to information and communication technologies is considered a social determinant of health, as it can generate inequalities in access to information and health services.

The editors believe that this Research Topic has advanced the understanding of the efficiency and welfare of an NHS, as well as how the healthcare system is related to the national economy and to a country's citizens.

## Author contributions

All authors listed have made a substantial, direct, and intellectual contribution to the work and approved it for publication.

## Conflict of interest

The authors declare that the research was conducted in the absence of any commercial or financial relationships that could be construed as a potential conflict of interest.

## Publisher's note

All claims expressed in this article are solely those of the authors and do not necessarily represent those of their affiliated organizations, or those of the publisher, the editors and the reviewers. Any product that may be evaluated in this article, or claim that may be made by its manufacturer, is not guaranteed or endorsed by the publisher.
